# Risky Behaviors among HIV-Positive Female Sex Workers in Northern Karnataka, India

**DOI:** 10.1155/2013/878151

**Published:** 2013-01-09

**Authors:** Apoorva Jadhav, Parinita Bhattacharjee, T. Raghavendra, James Blanchard, Stephen Moses, Shajy Isac, Shiva S. Halli

**Affiliations:** ^1^Population Studies Center, University of Pennsylvania, 3718 Locust Walk, 239 McNeil Building, Philadelphia, PA 19103, USA; ^2^Karnataka Health Promotion Trust, Bangalore 560044, India; ^3^Centre for Global Public Health, University of Manitoba, Winnipeg MB, Canada R3E 0T6

## Abstract

*Purpose*. Little is known about the risky sexual behaviors of HIV-positive female sex workers (FSWs) in the developing world, which is critical for programmatic purposes. This study aims to shed light on their condom use with regular clients as well as husband/cohabiting partner, a first in India. *Methods*. Multivariate logistic regression analyses for consistent condom use with regular clients and husband/cohabiting partner are conducted for the sample of 606 HIV-positive FSWs. *Results*. Older FSWs are 90% less likely and nonmobile FSWs are 70% less likely to consistently use condoms. FSWs on ART are 3.84 times more likely to use condoms. Additionally, FSWs who changed their occupation after HIV diagnosis are 70% less likely to use condoms. FSWs who are currently cohabiting are more likely to consistently use condoms with repeat clients and are 3.22 times more likely to do so if they have felt stigma associated with being HIV-positive. FSWs who have multiple repeat clients, and who do not know the sexual behavior of these clients, are more likely to use condoms consistently. *Conclusion*. This study would help inform programs to target the following particularly vulnerable HIV-positive FSWs: those who are older, those who changed their occupation post-HIV diagnosis, and those who are nonmobile.

## 1. Introduction

While overall HIV prevalence is low, India has the third largest number of people living with HIV/AIDS in the world [1]. Sexual transmission of HIV is the most dominant route of infection in the country and is concentrated among high risk groups, particularly female sex workers (FSWs), their clients, men who have sex with men (MSM), and injection drug users. Sentinel surveillance data from 2008-2009 indicates that 7.2% of injecting drug users, 7.4% of MSM, and 5.1% of FSWs are HIV-positive, in stark contrast to the 0.5% of attendees in antenatal clinics who tested positive [1]. These indicators suggest that the HIV epidemic in India is in its concentrated phase. FSWs have long been acknowledged as one of the primary drivers of sexually transmitted infections in developing countries [2, 3] and HIV in particular [4]. Due to the larger number of sexual partners compared to other populations, FSWs are at a higher risk of becoming infected and infecting their clients and other partners. Direct interventions among vulnerable groups like FSWs can prove to be very effective in India; Nagelkerke et al. [5] predict that effective HIV intervention among FSWs in the country can drive the epidemic to its extinction.

 National HIV prevalence among FSWs is estimated at 4.9%, but there is much geographical variation. Sentinel data indicate that at the state level, the highest HIV prevalence among FSWs is in Maharashtra (17.9%) and Manipur (13.1%), followed by Andhra Pradesh (9.7%), and Karnataka (5.3%) [6]. In addition to health problems, HIV-positive FSWs who know their HIV status are confronted with a multitude of decisions including those related to safe sex practices. Condom negotiation with clients and husband/cohabiting partner as well as consistent condom use regardless of type of client (regular, occasional, new) are complicated for FSWs as a whole, but take on a different dimension for FSWs who are HIV-positive. Although there are numerous studies conducted among FSWs regarding their risky sexual behaviors [2, 3, 7, 8], not a single study exits to the authors' knowledge on the nature of sex work practice among HIV-positive FSWs in India. The only other study that assesses these relationships finds that HIV-positive FSWs in China had more clients per month and used condoms consistently with regular partners than HIV-negative FSWs [9]. The sample size for that analysis was based on 47 HIV-positive FSWs in one city in China, thus was not generalizable to a larger population. In Karnataka, mapping exercises in 18 districts of the state indicate that there are 80,000 FSWs. If we assume an overall HIV prevalence of 15%, there will be 12,000 FSWs who are HIV-positive. Given the sheer number of HIV-positive FSWs in India, the findings of this study to understand condom use among FSWs and their regular clients and husband/cohabiting partner are particularly salient from a programmatic perspective. 

## 2. Materials and Methods

The data used for this study come from a cross-sectional quantitative survey of HIV-positive FSWs in three corridor districts in the south Indian state of Karnataka-Bagalkot, Belgaum, and Bijapur, conducted by the Karnataka Health Promotion Trust (KHPT). The data were collected for a period of 6 months, beginning from July to end of December 2011, and were analyzed in 2012. 

### 2.1. Sampling

The sampling frame for the study is a list of FSWs who tested positive for HIV in the study districts. The FSWs are referred to HIV testing by KHPT peer educators and are followed up to ensure they have visited the Integrated Counseling and Testing Center (ICTC) and undergone HIV testing. If the tested FSWs disclose their HIV status to the peer educators, they are recorded as part of the program monitoring system and followed up for services including pre-ART registration, CD4 tests, and ART treatment. The sample size for each study domain (district) was determined using the expected baseline value of 50% and a desired change in the baseline value of 10–15% with a design effect of 1.5. Based on this, the sample size was fixed at 339 HIV-positive FSWs per study domain. Additionally, assuming 10% nonresponse, the sample size was inflated to account for those instances. The final response rate was about 66%, which is high given the highly stigmatized population of study interest. Although the nonresponse rate of 33% is not desirable, it is to be noted that there were no significant differences by district especially in terms of their background characteristics except in their marital status, more *Devadasis* (*Devadasis* historically refer to a class of women who are ceremoniously married to gods or deities, and typically have socially sanctioned sexual liaisons with patrons. For a detailed discussion of the history and nuances of the tradition, refer to Srinivasan's 1985 piece “*Reform and Revival: The Devadasi and her Dance* [10]”) in Bagalkot district and more married sex workers in Belgaum district. These differences can be accounted for by controlling for the marital status variable. Hence, the analysis for this paper is performed for the pooled data of each domain separately so that there will be enough cases for both cross tabular and multivariate analyses. 

### 2.2. Selection of Respondents within a Domain (District)

The sample respondents were selected using stratified random sampling, with rural-urban residence as the first level, and *taluka *(an administrative subdivision consisting of towns and surrounding villages) as a second level of stratification. The age of respondents was considered as an implicit stratification variable so as to have a representative sample of the district. Once the list of HIV-positive FSWs was organized by stratification, a systematic random selection process was done to ensure that the sample respondents would be selected in proportion to the total number of positive FSWs in each stratum. The final sample size for completed interviews was 255 in Bagalkot district, 239 in Belgaum district, and 112 in Bijapur district for a total of size of 606 HIV-positive FSWs interviewed. 

### 2.3. Consent Process

The HIV status of FSWs is known only to the FSWs and peer educators, thus ensuring trust and communication between the two was critical. Prior to the survey, all the peer educators underwent a training program on the process, confidentiality, and expected roles and responsibilities. Additionally, the peer educators were the mediators who explained the objectives and expected outcome of the study with the HIV-positive FSWs. Only if the participating provided written and informed consent for the interview were they included in the final study interview process. 

### 2.4. Ethical Approval and Consent Process

Institutional review board at the St. John's Medical College, Bangalore, India, provided ethical approval for the study. The HIV status of the FSW is known only to the FSW and peer educators, thus ensuring trust and communication between the two was critical. Prior to the survey, all the peer educators underwent a training program on the process, confidentiality, and expected roles and responsibilities. Additionally, the peer educators were the mediators who explained the objectives and expected outcome of the study with the HIV-positive FSW. Only if the participating provided written and informed consent for the interview were they included in the final study interview process. 

### 2.5. Statistical Analysis

Once data editing, entry, and cleaning were completed by trained staff at KHPT, the data were analyzed using STATA, version 11.0. The frequencies and bivariate analysis were generated using this software. The multivariate analyses of consistent condom use with regular clients and husband/cohabiting partner were calculated along with 95% confidence intervals using logistic regression models.

## 3. Results

### 3.1. Sociodemographic Analysis

Of the 606 HIV-positive FSWs in the analysis, 93.6% were above age 25, with 46.5% between the ages of 25–34, and 47.0% above age 35. Three-fourths (73.1%) of the respondents were nonmobile (in these analyses, “mobile” FSWs refer to migrant or nonlocal women, and “nonmobile” refers to women who are local to the study area) residents and were predominantly Hindu (92.9%). As Table 1 shows, while most respondents were uneducated (82.5%), about 4.8% received more than 8 years of schooling. Sex work was not the sole occupation of a majority of respondents, with 66.7% engaged in another occupation. Additionally, 20% of respondents changed their occupation after a positive HIV diagnosis. There was no interdistrict variation between the respondents except for marital status, which is shown in Table 1. About 15% of respondents in Belgaum are married, compared to a much lower 2.4% in Bagalkot and 4.5% in Bijapur; 82% of respondents in Bagalkot are *Devadasi*, while 36.4% of respondents in Belgaum are widowed. Due to the lack of variation in most characteristics and small number of cases for each district, for subsequent multivariate analysis the data from all three districts are pooled with a control for district.

### 3.2. Sex Work and Condom Use

Of all the respondents, 19.9% move from their place of residence for sex work (Table 2). Of those, 13.2% travel every day, 53.7% travel once a week, while 21.5% travel multiple times in a week (Table 2). Most of the respondents were sex workers for duration of greater than 5 years; of those, 22.9% reported working between 5–9 years, while a much larger 64.7% reported working for greater than 10 years. Notably, the practice of sex work of FSWs was largely unknown to their husband/cohabiting partner, with 73.9% unaware about their partner's sex work. Interestingly, 47.7% of respondents believed that their husband/cohabiting partner has sexual relationships with other women, while 11.9% did not know. Multiple sexual partners were reported by the respondents themselves, however, with 56.6% of FSWs reporting having had sex with any nonpaying partner other than their husband/cohabiting partner. These nonpaying partners include police, pimps, and “special friends”. In terms of the nature of sex work, there was not much variation in number of clients per day, with a third reporting each: one, two, and three or more clients per day. In contrast, 18.6% of all respondents had one repeat client per day, while 40.1% had more than three repeat clients per day. The familiarity with repeat clients did not translate into knowledge of the number of sexual partners: 42.3% of the respondents did not know if the repeat clients had sex with other women. Finally, consistent condom use varied by type of partner: 32.9% with husband/cohabiting partner, 85% with nonpaying partner, and 73.3% with repeat clients. For multivariate analysis, we did not include occasional nonpaying partner as a dependent variable because the condom use among these clients was more than 75%, our cut-off for consideration for separate analysis.

### 3.3. Multivariate Analysis

The multivariate logistic regression results are conducted separately for two dependent variables: consistent condom use with husband/cohabiting partner (no/yes), and consistent condom use with repeat client (no/yes). Each model included controls for sociodemographic variables included in Table 1 and controls for district.

- Columns 2 and 3 of Table 3 refer to the logistic regression for consistent condom use with husband/cohabiting partner. The results indicate that older FSWs are 90% less likely to consistently use condoms compared to younger FSWs and nonmobile FSWs are 70% less likely to consistently use condoms compared to mobile FSWs. On the other hand, FSWs on ART are 3.84 times more likely to use condoms. Additionally, the number of clients per day is significant, with the odds of consistent condom use with husband/cohabiting partner increasing as the number of clients per day increases.
- Columns 4 and 5 of Table 3 refer to the logistic regression for consistent condom use with repeat clients. These results also find that nonmobile FSWs are less likely to use condoms than their mobile counterparts, and that FSWs who changed their occupation after HIV diagnosis are 70% less likely to use condoms that those who did not change their occupation. On the other hand, FSWs who are currently cohabiting are more likely to consistently use condoms with repeat clients, and are 3.22 times more likely to do so if they have felt any stigma associated with being HIV-positive. Notably, FSWs who have multiple repeat clients, and who do not know whether their repeat clients have sex with other women are significantly more likely to use condoms consistently.

## 4. Discussion

Other research in South India has found that inconsistent condom use with nonpaying partners or regular clients was based on the level of HIV risk perception by the FSWs [8]. Our study is unique in that the entire sample consists of HIV-positive FSWs, in order to better understand their risk-taking behaviors in light of their HIV status. Findings from this study indicate that older FSWs are less likely to consistently use condoms with their husband/cohabiting partner. This corresponds with the notion that older FSWs tend to be married and more dependent on their husband/cohabiting partner, so there is less negotiation in terms of condom use. This still indicates high-risk behavior since the husband/cohabiting partner does not always know the HIV status of his wife. 

While 12.2% of all respondents report consistent condom use with their husband/cohabiting partner when their HIV status is known to the partner, about 23.1% of FSWs do not consistently use condoms even though their HIV status is not known to their partner. Contrary to previous arguments [11], FSWs are less likely to consistently use condoms with their husband/cohabiting partner and repeat clients than mobile FSWs. Mobile FSWs are considered to be at higher risk of inconsistent condom use with their clients; however, as our findings show, it is the nonmobile FSWs who are less likely to consistently use condoms. Mobile sex workers tend to practice sex work in brothels or formalized set ups and are more likely to get support for condom negotiation with clients besides being more exposed to the program. The program activities include condom demonstration, condom negotiation with paying/nonpaying partners, and free supply of condoms in addition to empowerment exercises. It is notable that FSWs who changed their occupation after their HIV diagnosis are significantly less likely to use condoms consistently with their repeat clients. A possible explanation can be that these women are driven to sex work due to a drop in their income level, and are thus more vulnerable to the client's demands due to a potential source of income for the day.

The indicators that directly test the relationship between sex work and consistent condom use shed light on the nature of sex work in light of a positive HIV diagnosis for the FSWs. Those FSWs who are currently undergoing ART are more likely to consistently use condoms with their husband/cohabiting partner. FSWs undergoing any treatment regimen are generally more aware of the risks associated with unsafe sex, and the modes of transmission of HIV, thus are more likely to use condoms. It is interesting that this significant relationship only emerges for condom use with husband/cohabiting partner, and not for repeat clients. A sense of familiarity or belonging with a partner may then indicate lower risk taking behaviors. This is confirmed with our findings that as the number of clients per day increase, FSWs are more likely to use condoms with their husband/cohabiting partner. Another subset of FSWs who could require more focused attention are those FSWs who changed their occupation after HIV diagnosis, since they are less likely to consistently use condoms with repeat clients. This occupation change, lack of income, abandonment by spouse or families could indicate that these women may not be as aware of the risks involved with unsafe sex, or more importantly, may not be able to effectively negotiate condom use for fear of being turned away. A study on FSWs in Bangladesh finds that low rate of consistent condom use is a result of clients' dislike of condoms, lack of knowledge, low risk perception, and poor situational availability of condoms. Additionally, the authors find that initiation of condom negotiation by FSWs could trigger violence [7]. 

Notably, risky sex behavior is reduced with increasing numbers of repeat clients, and FSWs concern about other sexual relationships of their repeat clients. Known risk factors that contribute to the concentrated HIV epidemic in China, Thailand, and Cambodia are low rates of consistent condom use during sex work and concurrent sex with FSWs and other casual or steady partners [12]. The scenario in India is similar, thus targeted interventions for FSWs, particularly HIV-positive FSWs, in curtailing the spread of the epidemic are critical. 

Our study has several limitations. First, we do not have a comparison group of HIV-negative FSWs in order to assess the difference in risk taking behavior by HIV status. From what we know about HIV-negative FSWs, consistent condom use depends on the level of risk perception based on type of client. Those FSWs have to take appropriate actions to protect themselves in order to remain uninfected. In our study, the onus of responsibility is on the FSWs for another reason—in order to prevent infecting their husband/cohabiting partner or regular client. A second limitation is that questions were not asked on negotiation patterns of condom use. Thus, it is not possible to estimate risk perception on behalf of the clients, in light of knowing and not knowing the HIV status of the FSWs. Finally, we do not know about the alcohol use of clients or partners during sex, which can be correlated with limited condom negotiation. 

## 5. Conclusion

These findings are programmatically significant for interventions targeted at HIV-positive FSWs in Karnataka. Older FSWs, those who changed their occupation post-HIV diagnosis, and nonmobile FSWs are particularly vulnerable sub-populations that need pointed interventions. It is interesting to note that sizable proportion of sex workers do not inform their husband/cohabiting partner about their sex work or their HIV status. That means there are some high risk groups of sex workers additional to the ones we know. This information is extremely useful for the programmers working with HIV-positive FSWs in Karnataka. Similarly in case women fear going to program activities because they are HIV-positive and/or married, the program staff would greatly benefit from understanding the main factors that affect this decision and address these issues more effectively during counseling and other components of the intervention. For example, if women are unable to use condoms with their husband/cohabiting partner, the program staff could expand their work and include those partners in their counseling activities. Interventions should also include the family members in particular and communities in general in order to create a more enabling environment for safe sex practice for HIV-positive FSWs.

## Figures and Tables

**Table 1:** Sociodemographic distribution of HIV-positive FSWs in Northern Karnataka, 2011.

Category	Bagalkot (%)	Belgaum (%)	Bijapur (%)	Total (%)
Age				
<25 years	8.6	5.4	3.6	6.4
25–34 years	49.8	42.3	48.2	46.5
35+ years	41.6	52.3	48.2	47.0
Place of residence				
Mobile	23.5	33.9	19.6	26.9
Nonmobile	76.5	66.1	80.4	73.1
Religion				
Hindu	95.7	91.2	90.2	92.9
Others	4.3	8.8	9.8	7.1
Education				
None	82.3	81.6	84.8	82.5
1–7 years	11.4	15.1	10.7	12.7
8+ years	6.3	3.4	4.5	4.8
Marital status				
Married	2.4	15.1	4.5	7.8
Widowed	6.7	36.4	25.9	22.0
*Devadasi *	82.0	24.3	44.6	52.3
Others	9.0	24.3	25.0	18.0
Engaged in occupation besides sex work				
Yes	54.7	83.9	71.0	69.2
No	45.3	16.1	29.0	30.8
Changed occupation after diagnosis				
Yes	17.8	15.6	36.6	20.4
No	82.2	84.5	63.4	79.6
Currently on ART				
Yes	48.6	37.2	35.7	41.8
No	51.4	62.8	64.3	58.3
Duration of program exposure				
<2 years	8.7	22.7	2.7	13.1
3-4 years	21.0	47.9	40.2	35.2
5+ years	70.4	29.4	57.1	51.7

Number of respondents	253	238	112	603

**Table 2:** Nature of sex work and condom use among HIV-positive FSWs in Northern Karnataka, 2011.

Category	*N*	(%)
Mobility for sex work		
No	485	80.0
Yes	121	20.0
Frequency of mobility for sex work		
Everyday	16	13.2
Once a week	65	53.7
More than once a week	26	21.5
Fortnightly	9	7.4
Once a month	5	4.1
Usually under the influence of alcohol during sex work		
No	538	88.3
Yes	68	11.7
Duration in sex work		
<5 years	75	12.4
5–9 years	138	22.9
10+ years	391	64.7
Husband/cohabiting partner knows about sex work		
Yes	46	26.1
No	130	73.9
Husband/cohabiting partner has sexual relationships with other women		
Yes	84	47.7
No	71	40.3
Do not know	21	11.9
Ever had sex with any nonpaying partner other than husband/cohabiting partner		
Yes	343	56.6
No	263	43.4
Number of clients per day		
1	193	32.9
2	199	34.0
3+	194	33.1
Number of repeat clients		
1	63	18.6
2	140	41.3
3+	136	40.1
Does repeat client have sex with other women		
No	47	13.4
Yes	156	44.3
Do not know	149	42.3
Consistent condom use with husband/cohabiting partner		
No	120	67.0
Yes	59	33.0
Consistent condom use with repeat client		
No	94	26.7
Yes	258	73.3
Consistent condom use with nonpaying partner		
No	86	15.0
Yes	488	85.0

**Table 3:** Odds of consistent condom use with husband/co-habiting partner and repeat clients among HIV-positive FSWs in Northern Karnataka, 2011.

	Husband/Cohabiting Partner		Repeat Client	
	Odds Ratio	95% CI	Odds Ratio	95% CI
Age				
<25 years				
25–34 years	0.15	(0.02, 1.19)	0.65	(0.12, 3.54)
35+ years	0.10*	(0.01, 1.00)	0.77	(0.12, 4.81)
Place of residence				
Mobile				
Nonmobile	0.31*	(0.10, 0.96)	0.29*	(0.10, 0.93)
Marital status				
Married	—	—		
Widowed	—	—	2.43	(0.38, 15.48)
*Devadasi *	—	—	1.40	(0.22, 8.86)
Others	—	—	1.75	(0.26, 11.68)
Currently cohabiting				
Yes	—	—		
No	—	—	2.49*	(1.06, 5.89)
Currently on ART				
No				
Yes	3.84**	(1.39, 10.60)	1.36	(0.64, 2.86)
Feel any stigma associated with being HIV+				
No				
Yes	2.70	(0.94, 7.76)	3.22**	(1.34, 7.77)
Change in occupation after HIV diagnosis				
No	(ref)		(ref)	
Yes	0.35	(0.11, 1.13)	0.32**	(0.14, 0.72)
Number of clients per day				
1	(ref)		(ref)	
2	5.44*	(1.01, 29.26)	0.44	(0.17, 1.16)
3+	11.10***	(2.25, 54.68)	0.65	(0.23, 1.88)
Number of repeat clients				
1	—		(ref)	
2			1.31	(0.46, 3.75)
3+			3.23*	(1.09, 9.55)
Repeat client has sex with other women				
No			(ref)	
Yes			1.43	(0.49, 4.17)
Do not know			3.25*	(1.00, 10.53)

All models include controls for sociodemographic characteristics and controls for district.

*P < 0.05, **P < 0.01, ***P < 0.001.
